# Coronavirus Disease 2019 Infection as a Risk Factor for Infective Endocarditis

**DOI:** 10.7759/cureus.14813

**Published:** 2021-05-03

**Authors:** Dilesha Kumanayaka, Monica Mutyala, Dhinesh V Reddy, Jihad Slim

**Affiliations:** 1 Department of Internal Medicine, Saint Michael’s Medical Center/New York Medical College, Newark, USA; 2 Department of Medical Education, Saint Michael’s Medical Center/New York Medical College, Newark, USA; 3 Department of Infectious Diseases, Saint Michael’s Medical Center/New York Medical College, Newark, USA

**Keywords:** infective endocarditis, hypercoagulopathy, cytokine storms, sars-cov-2 infection, covid-19, covid-19 endocarditis, covid-19 coagulopathy side effects

## Abstract

Infective endocarditis (IE) is associated with relatively high morbidity and mortality and several risk factors have been identified in the past. Several predisposing factors for IE have been recognized in the literature, depending on the type of bacteria. Coronavirus disease 2019 (COVID-19) infection causes coagulopathy-associated complications and damage to many organ systems due to the inflammatory response induced by this viral infection. COVID-19 emerged only about a year ago and there are many unknown post-COVID-19 complications at this time. Here, we present the case of *Streptococcus mitis* IE in a patient with no prior predisposing factors other than diagnosis with COVID-19 a month ago.

## Introduction

Infection of the endocardial surface of the native valve, prosthetic heart valve, or an implanted cardiac device is known as infective endocarditis (IE). IE is a disease with relatively high morbidity and mortality [1-6]. Risk factors for IE usually involve two elements, the first is a transient bacteremia that could originate from dental infections, surgical procedures in the oral cavity, indwelling intravenous catheters, intravenous drug abuse, or infections of the skin, lungs, intestine, and urinary tract; the second is an abnormal valve either secondary to a congenital heart disease, degenerative disease, or prior surgical intervention. Commonly reported organisms causing IE include *Staphylococci*, *Streptococci*, *Enterococci*, and HACEK (*Haemophilus*, *Actinobacillus*, *Cardiobacterium*, *Eikenella*, and *Kingella*). *Staphylococcus aureus* has surpassed viridans streptococci as the most common cause of IE in recent years [3-6]. Mitral and aortic valves are the most commonly infected valves [3-6]. We present a unique case of IE caused by *Streptococcus mitis* on a structurally normal native mitral valve, where the only risk factor the patient had was a recent coronavirus disease 2019 (COVID-19) infection.

## Case presentation

**Figure 1:**
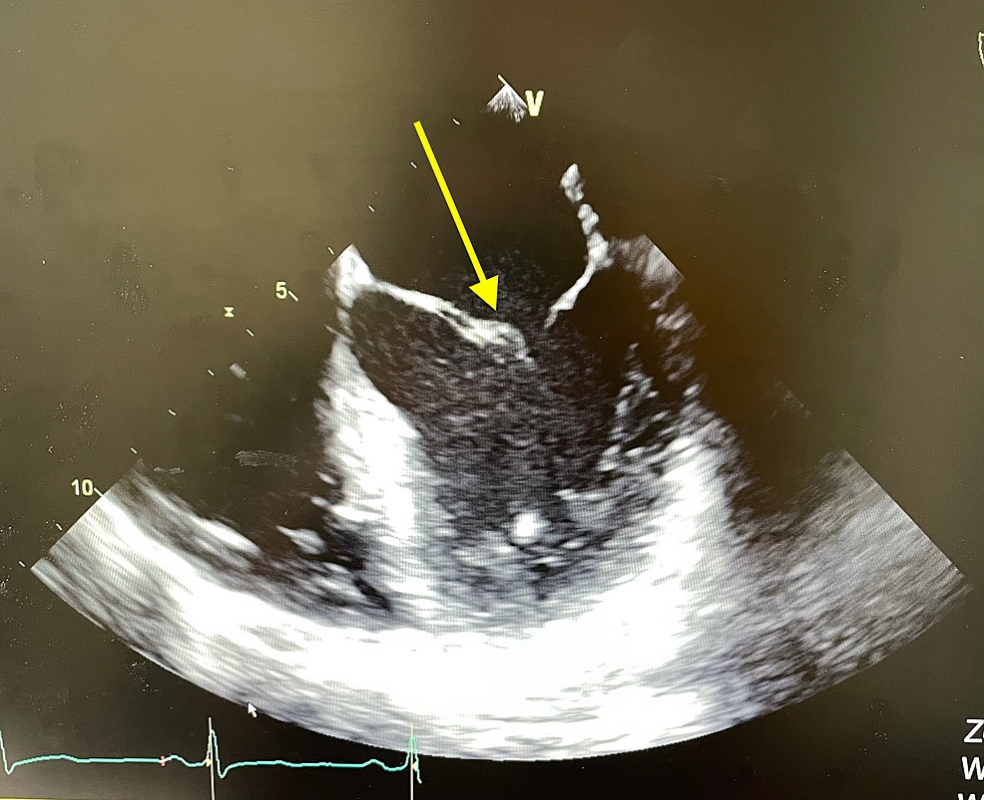
TEE showing a vegetation on the anterior mitral valve leaflet. TEE: transesophageal echocardiogram

A 38-year-old male with no known past medical history (PMH) presented with fevers/chills, night sweats, and periumbilical, non-radiating, and dull pain for about a week associated with anorexia and fatigue. He reported 20-pound weight loss in the prior month and had tested positive for COVID-19 a month earlier. According to the patient, he was hospitalized for four to five days with hypoxic episodes, requiring supplemental oxygen and received dexamethasone as well as anticoagulation. He did not have any risk factors for IE such as intravenous drug use, congenital heart disease, prosthetic heart valves, prior IE, or any recent dental procedure. On physical examination, he did not have any findings of heart murmurs and immunological/vascular skin findings. A complete septic workup was performed and demonstrated elevated erythrocyte sedimentation rate at 105 mm/hour (normal: 0-30 mm/hour), C-reactive protein at 15.6 mg/dL (normal: 0.0-0.8 mg/dL), white blood cells 11.4 × 10^3^/uL (normal 4.4-11 × 10^3^/uL), hemoglobin 12.2 × 10^3^/uL (normal: 13.5-17.5 × 10^3^/uL), and platelets 317 × 10^3^/uL (normal: 150-450 × 10^3^/uL). Urinalysis was normal and one set of blood cultures grew half bottles of *Streptococcus mitis*/*oralis*,with the next set of blood cultures a day apart showing no growth. A computed tomography scan of the abdomen was also done as the patient was complaining of pain and showed a 11 mm hypodensity in the spleen, suggesting an infarction. Transthoracic echocardiogram was performed, which could not exclude mitral valve endocarditis, but transesophageal echocardiogram (TEE) showed vegetation on the anterior mitral valve leaflet (Figure 1). He tested positive for COVID-19 IgG and negative for IgM. Antigen and polymerase chain reaction tests were also negative. He was discharged on ceftriaxone 2 g intravenously for six weeks via a PICC line and was followed up in the outpatient setting. His weekly labs were unremarkable and his repeat TEE did not show any vegetations.

## Discussion

Cardiovascular disease is the most common comorbidity associated with COVID-19 infection, as per the published literature to date. It is important to understand that severe COVID-19 disease can lead to serious long-term complications. Our patient had no significant PMH and no physical examination findings to suspect IE, but suspicion was high given the positive Duke’s score of one major criteria (TEE showing vegetation) and three minor criteria (pyrexia, vascular phenomena of splenic infarct, and one blood culture positive for *Streptococcus mitis*) [4-6]. In this case, as the patient did not have any risk factors for IE and had a history of hospitalization for severe COVID-19 disease, it can be suggested that the damage to the mitral valve structure by the cytokine storm with the systemic inflammation [1,2,7] and the hypercoagulable state induced by prior COVID-19 infection contributed as risk factors. The process of vegetation is initiated through a transient bacteremia, which causes organisms to adhere to the previously damaged endothelium [1,7]. The upregulated coagulation state by recent COVID-19 infection further helps microorganisms to encase in a platelet/fibrin matrix on the heart valve structure [1,7]. To the best of our knowledge, there have been no prior cases reported in the literature that proposes history of COVID-19 infection as a risk factor for future IE incidents.

## Conclusions

COVID-19 has been known to cause hyperinflammatory response along with hypercoagulable state leading to various complications. This case highlights the importance of considering prior COVID-19 infection as a possible risk factor for IE, especially in the absence of other predisposing factors, due to the organ damage that can result from inflammatory response and hypercoagulable state secondary to COVID-19 infection.

This case significantly benefits the fields of cardiology, infectious diseases, as well as other fields of internal medicine in the face of unprecedented challenges brought by a global pandemic. We hope that this novel case influences the approach to diagnosing IE in patients who have a history of COVID-19 infection. More studies that explore advantages of receiving treatment for COVID-19 infection in regards to post-disease complications would be beneficial.
